# Catalyst Influence on Undesired Side Reactions in the Polycondensation of Fully Bio-Based Polyester Itaconates

**DOI:** 10.3390/polym9120693

**Published:** 2017-12-09

**Authors:** Ina Schoon, Marcel Kluge, Steven Eschig, Tobias Robert

**Affiliations:** Fraunhofer Institute for Wood Research–Wilhelm-Klauditz-Institut WKI, Bienroder Weg 54E, 38108 Braunschweig, Germany; ina.schoon@wki.fraunhofer.de (I.S.); marcel.kluge@wki.fraunhofer.de (M.K.); steven.eschig@wki.fraunhofer.de (S.E.)

**Keywords:** bio-based polyester, itaconic acid, polyester, Ordelt-reaction, UV-curing materials

## Abstract

Bio-based unsaturated polyester resins derived from itaconic acid can be an alternative to established resins of this type in the field of radical-curing resins. However, one of the challenges of these polyester itaconates is the somewhat more elaborate synthetic process, especially under polycondensation conditions used on an industrial scale. The α,β-unsaturated double bond of the itaconic acid is prone to side reactions that can lead to the gelation of the polyester resin under standard conditions. This is especially true when bio-based diols such as 1,3-propanediol or 1,4-butanediol are used to obtain resins that are 100% derived from renewable resources. It was observed in earlier studies that high amounts of these aliphatic diols in the polyester lead to low conversion and gelation of the resins. In this work, a catalytic study using different diols was performed in order to elucidate the reasons for this behavior. It was shown that the choice of catalyst has a crucial influence on the side reactions occurring during the polycondensation reactions. In addition, the side reactions taking place were identified and suppressed. These results will allow for the synthesis of polyester itaconates on a larger scale, setting the stage for their industrial application.

## 1. Introduction

Over the last decades, the chemical industry and academia have made considerable efforts to replace petrochemical feedstock with building blocks derived from renewable resources [1,2,3,4,5,6,7]. However, bio-based building blocks can be more than a mere replacement for their petrochemical counterparts. A lot of these components are only accessible by biotechnological pathways, and have unprecedented structures that are not economically viable when synthesized by classical petrochemical routes [8]. Due to these new structures, bio-based building blocks not only have the potential to replace petrochemicals, but may also allow for new transformations and applications that can, in turn, lead to new materials with unprecedented properties [9].

In this respect, itaconic acid (IA) has drawn considerable attention over recent years. This α,β-unsaturated dicarboxylic acid has found applications as an alternative monomer for polyacrylates [4,10,11,12,13,14,15,16]. Recently, IA has also been extensively studied in the field of unsaturated polyesters [17,18,19,20,21,22]. This is not surprising, as polyesters of this type can find applications as bio-based thermal- or UV-curing resins, with applications including thermosets, coatings, printing inks, and materials for additive manufacturing. However, despite the research efforts in this field, polyester itaconates are not yet used on an industrial scale. Part of the problem is that many of the methods described in the literature are not compatible with processes conducted on an industrial scale. In addition, they may not possess optimal material properties.

**Scheme 1:**
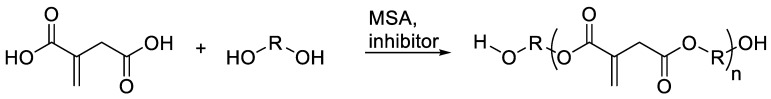
Condensation of polyester itaconates.

Over recent years, we have studied polyester itaconates for different applications, such as wood coatings [17,23] and printing inks [24]. In the course of these examinations, polyester itaconates were synthesized by reacting IA with different diols in the presence of methanesulfonic acid (MSA) as a catalyst. In addition, inhibitors were added to avoid thermally induced radical crosslinking during the polycondensation reaction (Scheme 1). This method is very similar to the polycondensation process conducted on an industrial scale; thus, upscaling to industrial quantities should be possible without tedious process optimizations and/or replacement of starting materials.

Under these reaction conditions, we obtained a range of polyester itaconates with different diols or polyols, such as 1,6-hexanediol, neopentylglycol, and glycerol. However, we encountered some unexpected difficulties in the course of synthesizing polyester itaconates that were completely derived from renewable resources: When 1,3-propanediol or 1,4-butanediol were used as diol components, the polycondensation suffered from lower conversions, even at longer reaction times. In addition, the reaction eventually resulted in the gelation of the polyester resin. This is somewhat unexpected, as other groups reported the use of these diols without any signs of crosslinking or gelation. However, in most of these cases, other catalytic systems that might reduce the risk of crosslinking were used. These systems included enzymes [25,26,27,28] or catalysts based on titanium [29,30,31] or tin [32,33,34]. In order to broaden the compatibility of our industry-related polycondensation method, a catalyst screening will be presented in this work. In addition, side reactions that are promoted by some catalysts will be examined and discussed.

## 2. Materials and Methods

### 2.1. Materials

Itaconic acid (99%) was purchased from ECEM (Amsterdam, The Netherlands). 1,3-Propanediol (purum) was received from DuPont Tate&Lyle (Loudon, TN, USA). 1,6-hexanediol (97%) was purchased from Dr. Lobinger Chemie (Seesen, Germany). 1,4-Butanediol (99%) and methanesulfonic acid (99%) were obtained from Carl Roth (Karlsruhe, Germany). Butylated hydroxytoluene (BHT, 99%) and 4-methoxyphenol (MeHQ, 98%), titanium butoxide (97%) and zinc acetate dihydrate (99%) were obtained from Sigma Aldrich (St. Louis, MO, USA). All reagents were used without further purification.

### 2.2. Measurements

The acid value (AV) is proportional to the unreacted acid groups. It was defined as the milligrams of potassium hydroxide required to neutralize one gram of sample, and was determined according to DIN EN ISO 2114 by titrating the carboxylic acid groups of the sample with potassium hydroxide solution in methanol (0.3 mol/L).

The hydroxyl value is defined as the number of milligrams of potassium hydroxide required to neutralize the acetic acid taken up on acetylation of one gram of a chemical substance that contains free hydroxyl groups. It was determined according to DIN EN ISO 4629-1. The method involved the acetylation of the hydroxyl groups in the sample with acetic anhydride in pyridine. After the acetylation, the remaining acetic anhydride was hydrolyzed with water and titrated with an aqueous potassium hydroxide solution (1 mol/L).

Infrared spectra were recorded by the ATR technique on a Thermo Scientific Nicolet iS5 FT-IR (Thermo Fisher Scientific, Waltham, MA, USA).

NMR experiments were conducted on a Bruker Avance III 400-MHz spectrometer (Bruker, Billerica, MA, USA) at 400 MHz for ^1^H NMR. Proton shifts are reported in ppm (δ) downfielded from tetramethylsilane (TMS), and were determined by reference to the residual solvent peak (CHCl_3_, 7.26 ppm for hydrogen atoms).

Determination of the molar mass distribution was performed by size exclusion chromatography (SEC) measurements on an Agilent 1200 Series with a variable UV-detector (here = 254 nm) and a refractive index detector (Agilent Technologies, Santa Clara, CA, USA). Tetrahydrofuran was used as an eluent with polystyrene calibration in the range of 162 to 70,000 g/mol. Three columns SDV 1000 A at 40 °C and the software (WinGPC Unity) were provided by Polymer Standard Service (Mainz, Germany).

### 2.3. Conversion

Conversion values were calculated based on the acid values. Therefore, a conversion of 0% corresponds to the theoretical AV determined by stoichiometric calculations at the beginning of the reaction. With the progress of the reaction, the AV decreases and the conversion rate increases. The data was used to build reaction kinetics.

### 2.4. Synthesis of Polyester Itaconates

Itaconic acid (1 eq.), diol (1.25 eq.), and inhibitors MeHQ (600 ppm) and BHT (800 ppm) were placed into a 500 mL three-necked round-bottom flask fitted with a Dean–Stark trap and a mechanical stirrer. The mixture was slowly heated to 130 °C, at which point toluene (30 mL) and the catalyst (0.4 wt %) were added. The mixture was then heated to 180 °C and kept at this temperature for the period indicated under constant reflux of the azeotropic solvent. The addition of the catalyst was considered as the start of the polycondensation reaction.

For the condensation reactions with an excess of 1,3-propanediol, the amount of the diol component was raised to 5.0 eq., whilst all other components remained constant.

## 3. Results and Discussion

**Scheme 2:**
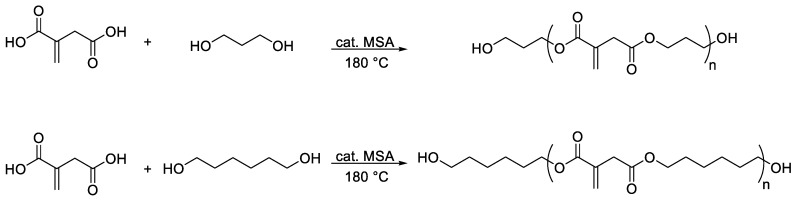
Polycondensation of Itaconic Acid (IA) with 1,3-propanediol (PDO) and 1,6-hexanediol (HDO).

In the course of our studies of completely bio-based polyester itaconates, it was observed that a high amount of bio-based 1,3-propanediol (PDO) or 1,4-butanediol (BDO) in the polycondensation reactions with itaconic acid (IA) leads to lower conversions, as well as gelation of the reaction mixture. However, this was not observed when 1,6-hexanediol (HDO) was used. In order to understand the incompatibility of the bio-based diols in this reaction, we first compared two polycondensation reactions of IA with PDO, and IA with HDO. The reactions were conducted with a diol:diacid ratio of 1.25:1.0 in the presence of methanesulfonic acid (MSA), which was used as a catalyst (Scheme 2).

**Figure 1:**
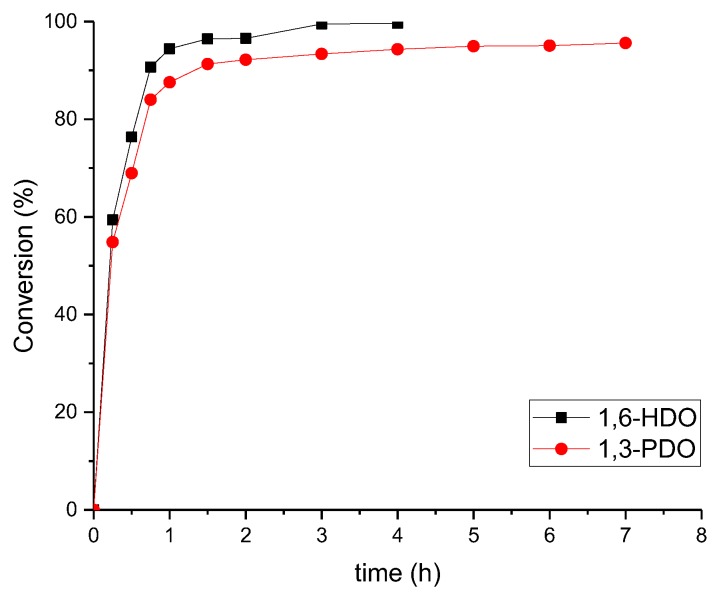
Conversion of the polycondensation reaction of IA with HDO (**squares**) and PDO (**circles**).

The conversion was followed by measuring the acid value at the beginning of the reaction, every 15 min, and later every 30 or 60 min. Figure 1 shows the conversion of both reactions over time.

In the case of HDO, the conversion was virtually complete (>99%) after 3 h at 180 °C, yielding a polyester resin with low viscosity and with no sign of crosslinking or gelation. In addition, the resin was completely soluble in acetone. When PDO was used as a diol, the reaction was slower, with a conversion of 95% after 6 h. However, after this, the conversion stagnated and the reaction mixture gelled after 7 h, resulting in a highly cross-linked material that was no longer soluble in acetone or other solvents.

In order to circumvent this problem, and understand the side reactions occurring during this polycondensation process, a catalyst screening for the reaction of IA with PDO was undertaken. The catalysts used included MSA, Zn(OAc)_2_, and Ti(OBu)_4_. Besides the experiment shown in Figure 1, another experiment with MSA was conducted. This involved the addition of MSA to the reaction mixture after 6 h. Due to the water sensitivity of Ti(OBu)_4_, this catalyst was also added after 6 h. At this time, most of the theoretical amount of water was collected in the Dean–Stark trap. In addition, one experiment without catalyst was conducted as a control experiment. An overview of the experiments is given in Table 1.

**Figure 2:**
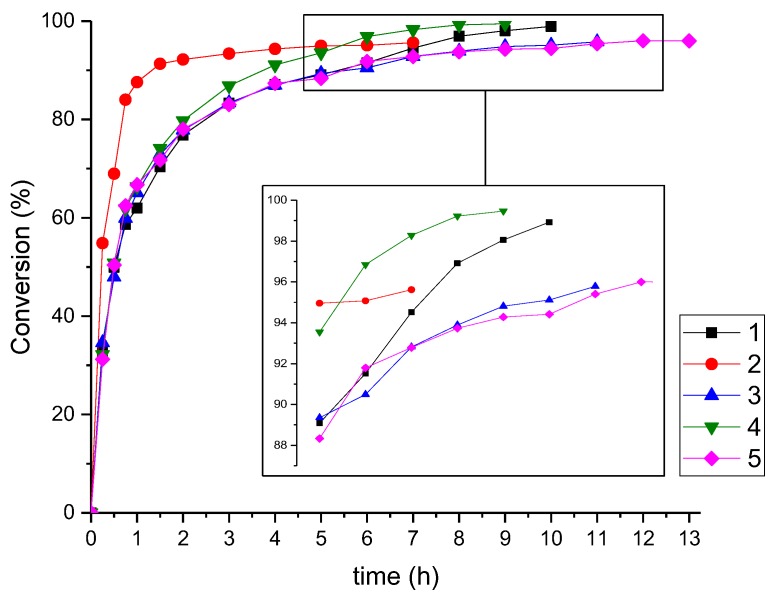
Conversion of the polycondensation of IA with PDO in the presence of different catalysts (see Table 1).

As depicted in Figure 2, the choice of catalyst has a crucial effect on the polycondensation reaction. In the first 4 h of the reaction, the use of MSA (Curve 2) showed the highest rate of conversion, with more than 90% converted in less than 2 h. However, the conversion stagnated after 2 h, and leveled off at around 95% after 4 h. In addition, the reaction mixture gelled after 7 h of reaction time. With no catalyst added, the polycondensation rate was slower, leading to a conversion of 96% after 13 h. When MSA was added after 6 h, the polycondensation rate only increased marginally, despite the addition of the catalyst. However, it also led to the gelation of the reaction mixture after 11 h. Condensations with the other catalysts showed similar conversion rates in the first 3 h. However, the use of Zn(OAc)_2_ resulted in a further increase to more than 99% conversion after 9 h reaction time (Curve 4), without any sign of crosslinking or gelation. The addition of Ti(OBu)_4_ after 6 h also led to high conversion of 98% after 9 h without any observed cross-linking. However, due to the late addition of the catalyst, the overall reaction progress was slower. Table 1 shows the conversion of the polyesters synthesized in the presence of different catalysts after a reaction time of 9 h.

**Figure 3:**
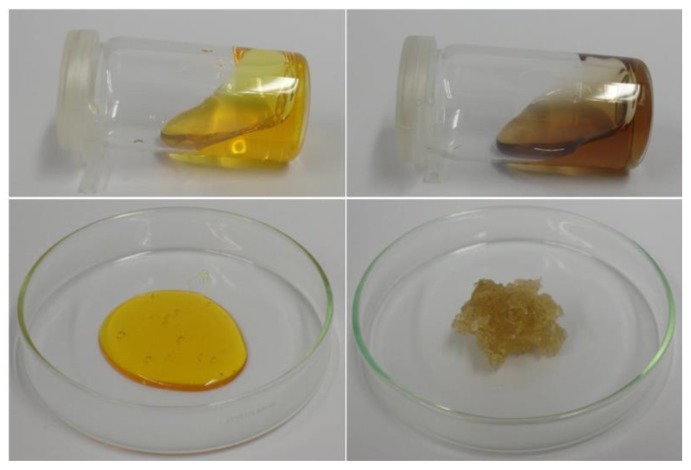
Digital photographs of the polyester resins obtained in the presence of Zn(OAc)_2_ (**left**) and MSA (**right**) after 6 h (**upper row**), and at the end of the reaction (**lower row**).

Figure 3 shows samples taken from the polycondensation reaction with Zn(OAc)_2_ (left) and MSA (right) after 6 h (upper row), and at the end of the reaction (lower row). The use of Zn(OAc)_2_ yielded a viscous resin, whereas an unusable gelled polymer was obtained when MSA was used as catalyst.

The gelation of the polycondensation in the presence of MSA is a strong indication that a side reaction is taking place. Initially, a radical crosslinking reaction was considered as the reason for this gelation. However, the same amounts of inhibitors were used in all reactions. In addition, the gelation did not occur when HDO was used as diol component. Therefore, a radical crosslinking reaction pathway can be excluded as the underlying cause of gelation. Another possible cause of the crosslinking is the nucleophilic addition of alcoholic hydroxyl groups (oxa-Michael Addition) to the α,β-unsaturated double bond of the itaconic acid moiety. This reaction, also known as the Ordelt reaction, was studied extensively in the 1960s in conventional unsaturated polyester resins [35,36,37,38,39].

**Scheme 3:**
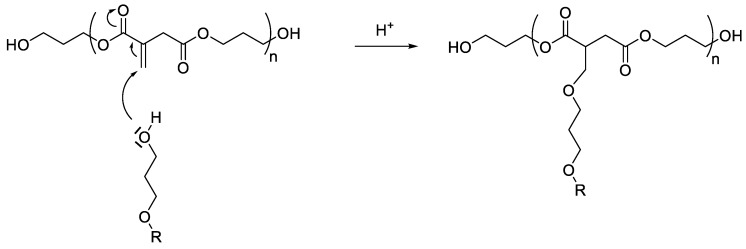
Mechanism of the oxa-Michael Addition (Ordelt reaction) of alcoholic hydroxyl groups, resulting in the crosslinking of the polyester (R = polymer chain).

Recently, this reaction was examined in more detail by Farmer et al. in the case of polyesters derived from itaconic acid (Scheme 3) [31]. They found that in their case, when itaconic acid esters were used, less than 4% of the diol was added to the unsaturated double bond. However, it was shown by Kharas et al. that the degree of Ordelt reaction increases with the acidity of the catalyst [40]. This would explain why the gelation is only observed in the case of the stronger Brønsted-acid MSA.

The crosslinking of the polymer chains would lead to larger polymer networks and, in turn, to polymers with higher molecular weights. However, this is not detectable by means of size exclusion chromatography (SEC). A comparison of the SEC traces of polyesters synthesized with MSA and Zn(OAc)_2_ after a 6 h reaction time does not reveal any significant difference between the two samples (see Supporting Materials, Figures S1–S3). In both cases, only a small amount of high molecular weight polyester can be observed. Unfortunately, SEC measurements of the gelled polyester were not possible, as the material was no longer soluble.

**Figure 4:**
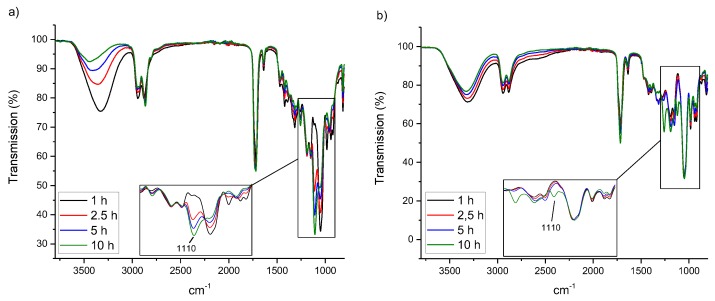
FT-IR spectra of the polycondensation of IA with an excess of PDO in the presence of (**a**) MSA and (**b**) Zn(OAc)_2_.

In order to elucidate if the Ordelt reaction is indeed responsible for the crosslinking and gelation of the polyesters, a test reaction was performed. For this, itaconic acid was reacted with a 5-fold excess of PDO in the presence of MSA, and in a second experiment in the presence of Zn(OAc)_2_. The reaction was monitored via FT-IR by taking samples every 30 min. The C=C stretch around 1640 cm^−1^ and the C=C deformation vibration should decrease over time, as the C=C double bonds will slowly be consumed by the nucleophilic attack of the hydroxyl group. However, during the course of both reactions, only a slight decrease of the C=C vibrations could be observed. On the other hand, it became apparent that the use of MSA as a catalyst resulted in a significant decrease in the broad O–H stretch vibration signal around 3400 cm^−1^, and the C–O stretch vibration around 1050 cm^−1^. This suggests a greater loss of OH groups during the polycondensation reaction compared to the experiment with Zn(OAc)_2_ (Figure 4). As the PDO was present in a 5-fold excess, the decline cannot be explained by the esterification reaction. In addition, evaporation of the PDO during the polycondensation process can also be excluded as the main reason for the loss of OH functionalities, as this effect would be equal for both reactions. As the C=C double bond does not decrease significantly at the same time, the only plausible explanation of this phenomenon is the formation of ether linkages. This process seems to be a major side reaction when MSA is utilized as the catalyst. This theory is supported by the emergence of a signal at around 1110 cm^−1^. This corresponds to the C–O stretch vibration of an ether, and is already very pronounced after 2.5 h. Again, this vibration is negligible when Zn(OAc)_2_ is used, and only appears as a small signal after 10 h at 1120 cm^−1^.

**Figure 5:**
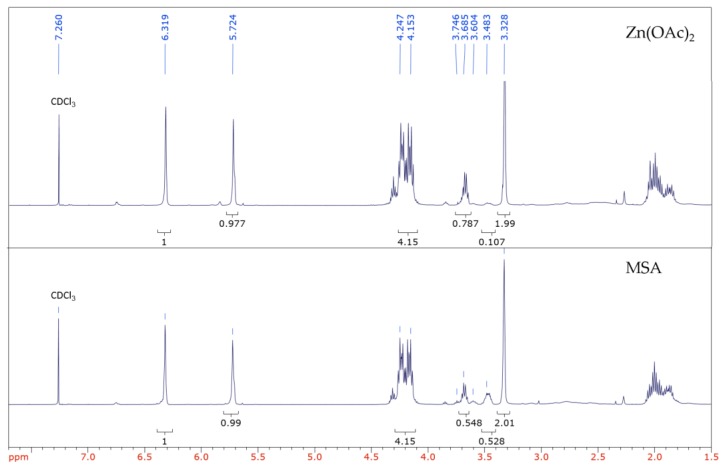
^1^H NMR of the polycondensation reaction with MSA (**below**) and Zn (OAc)_2_ (**top**) in CDCl_3_.

The difference in ether formation was also be confirmed by means of NMR spectroscopy. Figure 5 shows the ^1^H NMR of the polycondensation reactions 2 and 4 (Table 1) after 7 h at 180 °C in CDCl_3_. The use of MSA resulted in the appearance of a signal at 3.47 ppm, which corresponds to with the O–CH_2_ of the ether linkages. This signal was far less pronounced when Zn(OAc)_2_ was used as a catalyst. In addition, there was a difference in the signal intensity of the terminal OH groups around 3.75. Signals associated with addition products of an Ordelt reaction are difficult to detect and assign. Farmer et al. suspected the signals of O–CH_2_ groups of the addition products to appear at around 3.71 and 3.55 ppm [31], which seem to be less pronounced when Zn(OAc)_2_ is used. Again, due the gelation of the condensation reaction with MSA, NMR measurements of the gelled polyester were not possible.

To further confirm this hypothesis, the OH values of the experiments 1–5 (Table 1) were measured and compared with the theoretical OH values (OHV) (Table 2). When no catalyst was used (entry 5), the difference between the theoretical and the determined OHV showed a discrepancy of 19 mg KOH/g. As side reactions can be neglected when no catalyst is present, the difference can be explained by a loss of PDO due to evaporation during the polycondensation process. When MSA was applied from the start of the reaction, the gelation after 7 h made the determination of the OHV infeasible. However, after the addition of MSA after 6 h, the highest deviation of 100 mg KOH/g was observed. This is an indication of a high degree of side reaction. In the case of Zn(OAc)_2_ and Ti(OBu)_4_, the deviation of the OHV was less pronounced, with a difference of 36 and 52 mg KOH/g. This is an indication that when these Lewis acids were used, side reactions were taking place to a lesser degree, perhaps explaining why gelation was not observed in this case.

**Figure 6:**
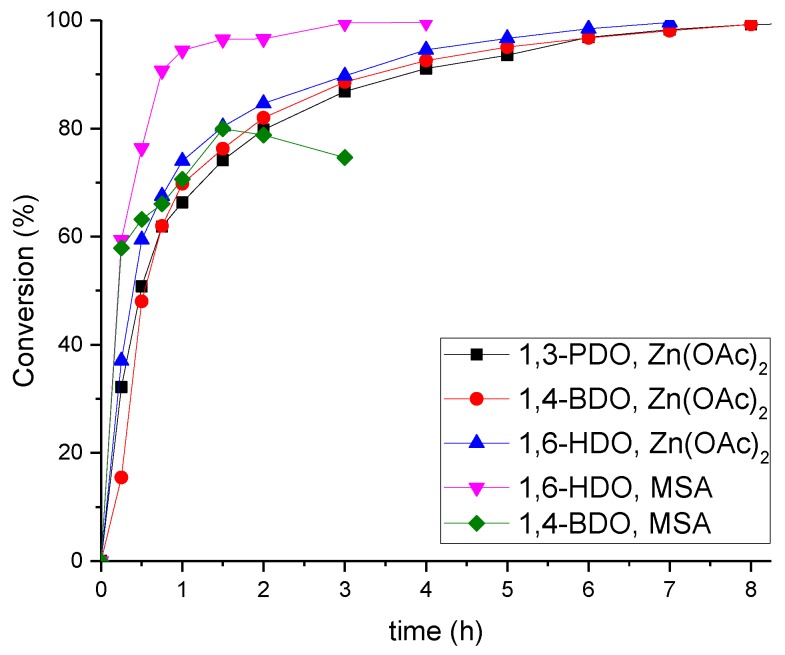
Conversion curves of the polycondensation reactions of itaconic acid with 1,3-propanediol, 1,4-butanediol, and 1,6-hexanediol in the presence of MSA or Zn(OAc)_2_.

We therefore conclude that the gelation is a result of the combination of etherification and Ordelt reaction. The etherification leads to a deviation in the ratio of diols and diacids, and therefore higher molecular weights of the polyester. In combination with the undesired crosslinking through Ordelt reactions, the polyesters eventually form large networks, resulting in gelation of the reaction mixture [41,42,43]. Given this hypothesis, further diols were tested. Of special interest in this case was BDO, as polyesters based on the reaction of this diol with IA also result in gel formation. We therefore followed the reaction kinetics of BDO with MSA and Zn(OAc)_2_. In addition, the polycondensation of HDO with IA was studied in the presence of Zn(OAc)_2_. Figure 6 shows the conversion curves of these reactions. For comparison, Figure 6 also shows the polycondensation of PDO and IA with Zn(OAc)_2_, and HDO with IA in the presence of MSA.

The most striking result was the course of the conversion curve of the reaction between BDO and MSA. Already after 30 min, the conversion had slowed down significantly, and by 90 min had decreased. Even though this might be counter-intuitive, the reason for this effect is quite straightforward. As for PDO, MSA also favors the etherification with BDO [38,39]. However, BDO undergoes an intramolecular ether formation to yield tetrahydrofuran (THF). This, in turn, results in the evaporation of the THF being formed, and therefore a loss in mass of the reaction mixture. As the conversion is estimated via the AV of the reacting mixture, and is specified as mg KOH/g, the number of acid groups per gram of sample rises again. Therefore, MSA is not suitable as a catalyst for the polycondensation of BDO under these conditions. However, if Zn(OAc)_2_ was used, the polycondensation reaches a conversion of >99% after 8 h with no significant difference to the other diols: PDO and HDO. The condensation of the latter was slower than the reaction with MSA, but a conversion of >99% was obtained after 7 h. Therefore, Zn(OAc)_2_ can also be used as catalyst for polycondensations of BDO, and circumvents the problem of THF formation during the reaction.

## 4. Conclusions

Polycondensation reactions used to synthesize polyester resins for coating applications are well understood, and have been used for decades on an industrial scale. However, there are still challenges that need to be addressed. This is especially true when new bio-based building blocks come into play that possess properties that are considerably different from established monomers derived from petrochemical feedstock. In this work, we showed that polycondensation reactions with itaconic acid under industry-relevant conditions can result in problems not occuring other (unsaturated) polyester synthesis. It was shown that Brønsted acids, such as MSA, are an inadequate choice of catalyst for polycondensation reactions with diols that are prone to etherification as a competing side reaction. An improved catalytic system was identified by conducting a catalyst screening with different catalysts and methods of addition. The water-tolerant Lewis acid Zn(OAc)_2_ was found to be the catalyst of choice for the polycondensation reaction of itaconic acid, especially with the bio-based diols 1,3-propanediol and 1,4-butanediol. Despite the somewhat slower reaction kinetics compared to MSA, the desired products can be obtained with a conversion of >99% after 9 h with no sign of gelation of the desired polyester. This study shows that replacing petrochemical starting materials with compounds from renewable resources can result in unforeseeable and undesired side reactions. This, in turn, makes modification of established synthetic procedures necessary. Therefore, the method described in this study can help to further increase the amount of bio-based monomers used on an industrial scale, resulting in more products that are 100% derived from renewable resources.

## Figures and Tables

**Table 1:** Conversion of the catalyst screening of the polycondensation of IA with PDO.

Polyester	Catalyst	Conversion after 9 h
1	Ti(OBu)_4_^a^	98%
2	MSA	96% ^b^
3	MSA ^a^	96% ^c^
4	Zn(OAc)_2_	>99%
5	no catalyst	94%

^a^ Addition of the catalyst after 6 h; ^b^ conversion after 7 h due to gelation of the polyester; ^c^ gelation of the polyester after 11 h.

**Table 2:** Theoretical and experimental OHV of the different polycondensation experiments.

Polyester	Catalyst	t/h	Theoretical OHV [mg KOH/g]	Experimental OHV [mg KOH/g]	Δ OHV [mg KOH/g]
1	Ti(OBu)_4_^a^	10	153	101	52
2	MSA	7	169	- ^b^	-
3	MSA ^a^	11	168	68	100
4	Zn(OAc)_2_	9	151	115	36
5	No catalyst	13	166	147	19

^a^ Addition of the catalyst after 6 h; ^b^ due to the gelation of the polyester, the determination of the OH value (OHV) was not possible.

## Supplementary Materials

The following are available online at www.mdpi.com/2073-4360/9/12/693/s1. Figure S1: SEC traces of the polycondensation reaction of itaconic acid with 1,3-propanediol in the presence of MSA as catalyst; Figure S2: SEC traces of the polycondensation reaction of itaconic acid with 1,3-propanediol in the presence of Zn(OAc)2 as catalyst; Figure S3: Comparison of the SEC traces of the polycondensation reaction of itaconic acid with 1,3-propanediol after 7.5 h in the presence of MSA and Zn(OAc)_2_.
