# Supplementary material for: Catalyst Influence on Undesired Side Reactions in the Polycondensation of Fully Bio-Based Polyester Itaconates
Source: Polymers (Basel). 2017 Dec 9;9(12):693. doi: 10.3390/polym9120693 (PMC6418628; doi:10.3390/polym9120693)
Supplement: Supplementary file 1 [file polymers-09-00693-s001.pdf]

polymers

# Catalyst influence on undesired side-reactions in the polycondensation of fully bio-based polyester itaconates

**Ina Schoon , Marcel Kluge, Steven Eschig, and Tobias Robert***

Fraunhofer Institute for Wood Research – Wilhelm-Klauditz-Institut WKI, Bienroder Weg 54E, 38108 Braunschweig, Germany. E-mail: tobias.robert@wki.fraunhofer.de
* Correspondence: tobias.robert@wki.fraunhofer.de; Tel.: +49-531-2155-357

Academic Editor: name
Received: date; Accepted: date; Published: date

## Supporting Information

Size Exclusion Chromatography (SEC) Measurements

Determination of the molar mass distribution was performed by SEC measurements with tetrahydrofuran as eluent and with polystyrene-calibration in the range of 162 g/mol to 70,000 g/mol. Three columns SDV 1000A at 40◦C, a variable UV-detector (here: 254 nm), a refractive index detector and the software (WinGPC Unity) were provided by Polymer Standard Service (Mainz, Germany). Samples of the polycondensation reaction of Itaconic acid with 1,3-propanediol in the presence of methanesulfonic acid (MSA) and zinc acetate ($Zn(OAc)_2$) were taken after 6.5, 7, and 7.5 hours. The molecular weight distribution for the polycondensation reaction with MSA is shown in figure S1, for $Zn(OAc)_2$ in figure S2. In addition, figure S3 shows the molecular weight distribution of both reaction at 7.5 h.

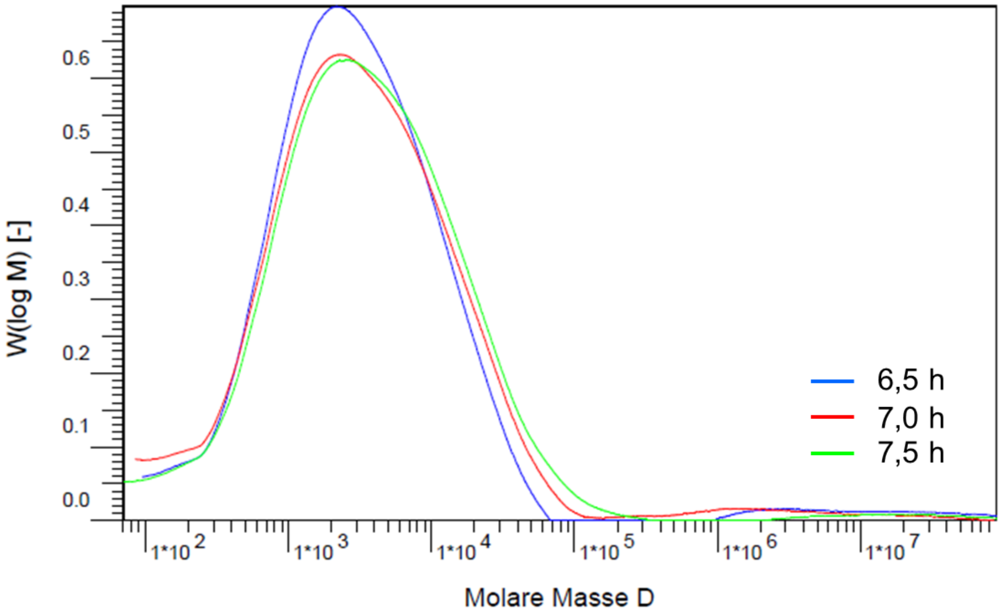


**Figure S1**. SEC traces of the polycondensation reaction of itaconic acid with 1,3-propanediol in the presence of MSA as catalyst.

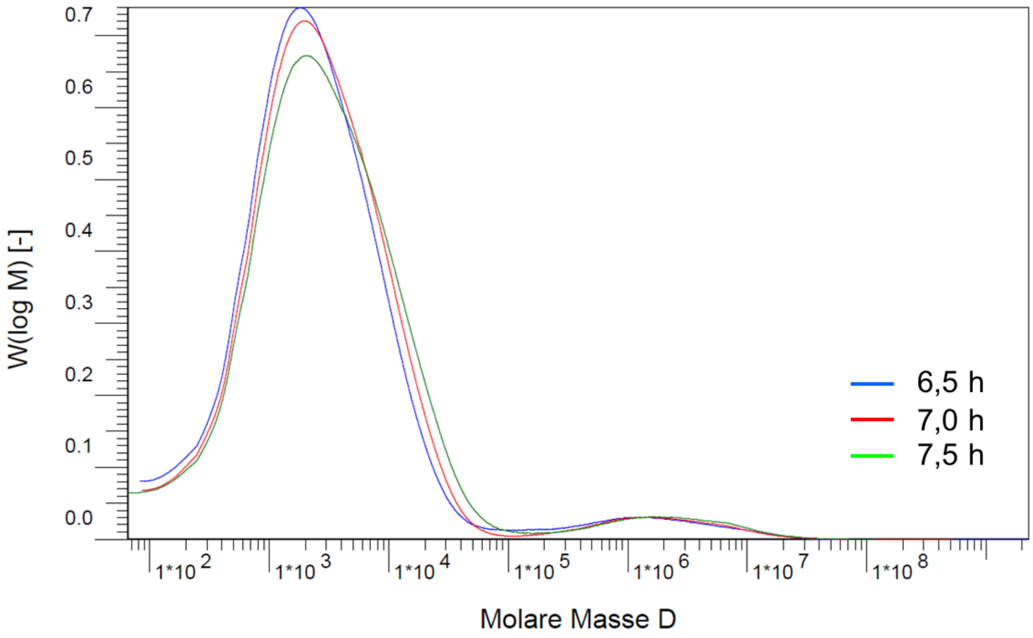


**Figure S2**. SEC traces of the polycondensation reaction of itaconic acid with 1,3-propanediol in the presence of $Zn(OAc)_2$ as catalyst.

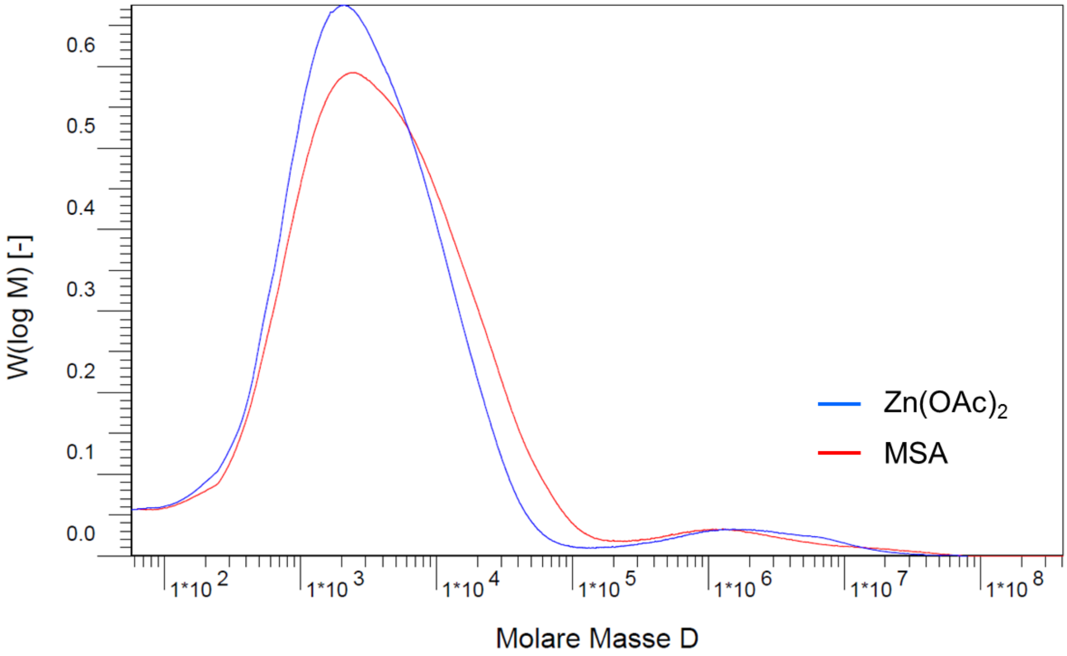


**Figure S3**. Comparison of the SEC traces of the polycondensation reaction of itaconic acid with 1,3-propanediol after 7.5 h in the presence of MSA and $Zn(OAc)_2$ .
